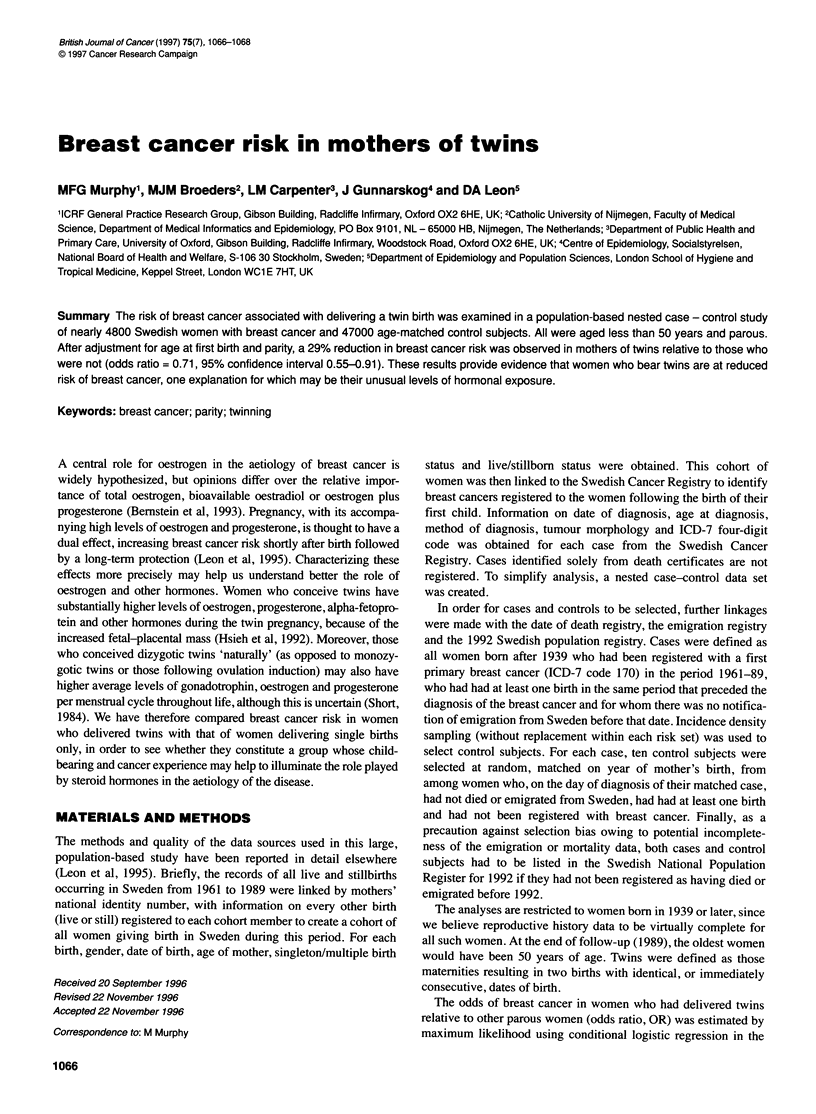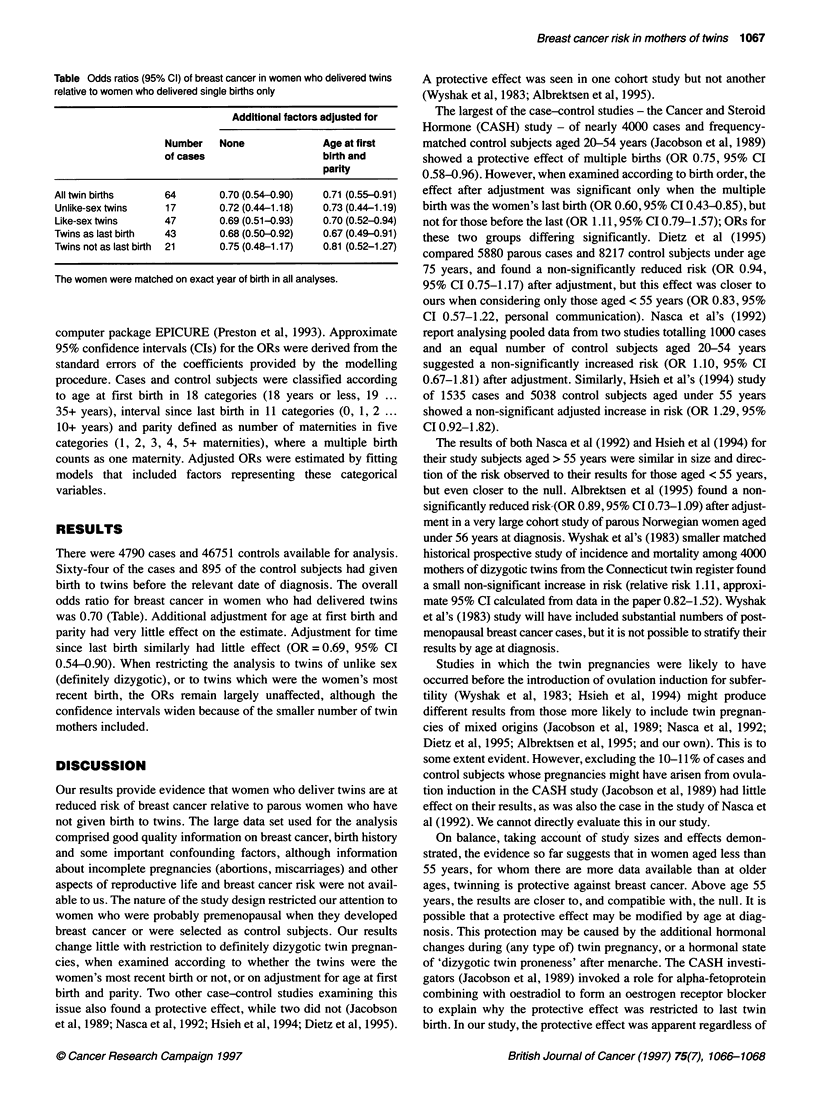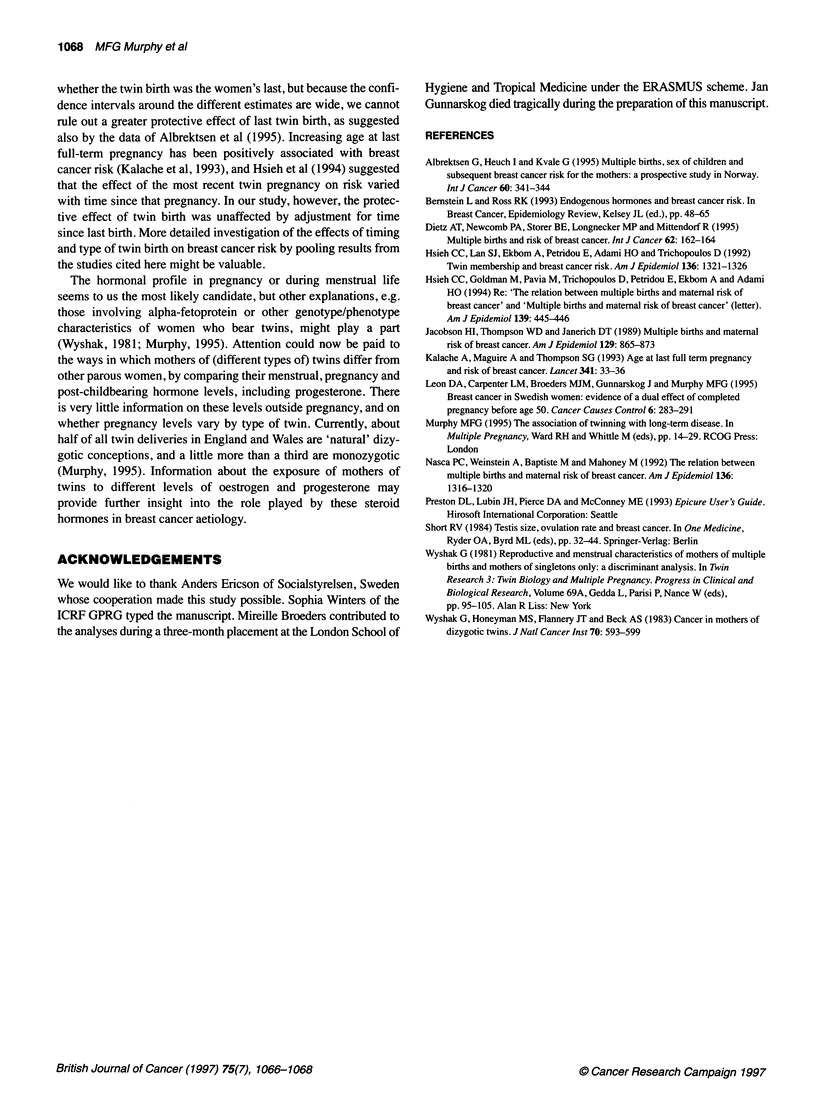# Breast cancer risk in mothers of twins.

**DOI:** 10.1038/bjc.1997.181

**Published:** 1997

**Authors:** M. F. Murphy, M. J. Broeders, L. M. Carpenter, J. Gunnarskog, D. A. Leon

**Affiliations:** ICRF General Practice Research Group, Radcliffe Infirmary, Oxford, UK.

## Abstract

The risk of breast cancer associated with delivering a twin birth was examined in a population-based nested case-control study of nearly 4800 Swedish women with breast cancer and 47000 age-matched control subjects. All were aged less than 50 years and parous. After adjustment for age at first birth and parity, a 29% reduction in breast cancer risk was observed in mothers of twins relative to those who were not (odds ratio = 0.71, 95% confidence interval 0.55-0.91). These results provide evidence that women who bear twins are at reduced risk of breast cancer, one explanation for which may be their unusual levels of hormonal exposure.


British Journal of Cancer (1997) 75(7), 1066–1068
© 1997 Cancer Research Campaign

### Breast cancer risk in mothers of twins

**MFG Murphy[1], MJM Broeders[2], LM Carpenter[3], J Gunnarskog[4] and DA Leon[5]**

[1]ICRF General Practice Research Group, Gibson Building, Radcliffe Infirmary, Oxford OX2 6HE, UK; [2]Catholic University of Nijmegen, Faculty of Medical Science, Department of Medical Informatics and Epidemiology, PO Box 9101, NL – 65000 HB, Nijmegen, The Netherlands; [3]Department of Public Health and Primary Care, University of Oxford, Gibson Building, Radcliffe Infirmary, Woodstock Road, Oxford OX2 6HE, UK; [4]Centre of Epidemiology, Socialstyrelsen, National Board of Health and Welfare, S-106 30 Stockholm, Sweden; [5]Department of Epidemiology and Population Sciences, London School of Hygiene and Tropical Medicine, Keppel Street, London WC1E 7HT, UK

**Summary** The risk of breast cancer associated with delivering a twin birth was examined in a population-based nested case – control study of nearly 4800 Swedish women with breast cancer and 47000 age-matched control subjects. All were aged less than 50 years and parous. After adjustment for age at first birth and parity, a 29% reduction in breast cancer risk was observed in mothers of twins relative to those who were not (odds ratio = 0.71, 95% confidence interval 0.55–0.91). These results provide evidence that women who bear twins are at reduced risk of breast cancer, one explanation for which may be their unusual levels of hormonal exposure.

**Keywords:** breast cancer; parity; twinning

A central role for oestrogen in the aetiology of breast cancer is widely hypothesized, but opinions differ over the relative importance of total oestrogen, bioavailable oestradiol or oestrogen plus progesterone (Bernstein et al, 1993). Pregnancy, with its accompanying high levels of oestrogen and progesterone, is thought to have a dual effect, increasing breast cancer risk shortly after birth followed by a long-term protection (Leon et al, 1995). Characterizing these effects more precisely may help us understand better the role of oestrogen and other hormones. Women who conceive twins have substantially higher levels of oestrogen, progesterone, alpha-fetoprotein and other hormones during the twin pregnancy, because of the increased fetal–placental mass (Hsieh et al, 1992). Moreover, those who conceived dizygotic twins 'naturally' (as opposed to monozygotic twins or those following ovulation induction) may also have higher average levels of gonadotrophin, oestrogen and progesterone per menstrual cycle throughout life, although this is uncertain (Short, 1984). We have therefore compared breast cancer risk in women who delivered twins with that of women delivering single births only, in order to see whether they constitute a group whose child-bearing and cancer experience may help to illuminate the role played by steroid hormones in the aetiology of the disease.

### MATERIALS AND METHODS

The methods and quality of the data sources used in this large, population-based study have been reported in detail elsewhere (Leon et al, 1995). Briefly, the records of all live and stillbirths occurring in Sweden from 1961 to 1989 were linked by mothers' national identity number, with information on every other birth (live or still) registered to each cohort member to create a cohort of all women giving birth in Sweden during this period. For each birth, gender, date of birth, age of mother, singleton/multiple birth status and live/stillborn status were obtained. This cohort of women was then linked to the Swedish Cancer Registry to identify breast cancers registered to the women following the birth of their first child. Information on date of diagnosis, age at diagnosis, method of diagnosis, tumour morphology and ICD-7 four-digit code was obtained for each case from the Swedish Cancer Registry. Cases identified solely from death certificates are not registered. To simplify analysis, a nested case–control data set was created.

In order for cases and controls to be selected, further linkages were made with the date of death registry, the emigration registry and the 1992 Swedish population registry. Cases were defined as all women born after 1939 who had been registered with a first primary breast cancer (ICD-7 code 170) in the period 1961–89, who had had at least one birth in the same period that preceded the diagnosis of the breast cancer and for whom there was no notification of emigration from Sweden before that date. Incidence density sampling (without replacement within each risk set) was used to select control subjects. For each case, ten control subjects were selected at random, matched on year of mother's birth, from among women who, on the day of diagnosis of their matched case, had not died or emigrated from Sweden, had had at least one birth and had not been registered with breast cancer. Finally, as a precaution against selection bias owing to potential incompleteness of the emigration or mortality data, both cases and control subjects had to be listed in the Swedish National Population Register for 1992 if they had not been registered as having died or emigrated before 1992.

The analyses are restricted to women born in 1939 or later, since we believe reproductive history data to be virtually complete for all such women. At the end of follow-up (1989), the oldest women would have been 50 years of age. Twins were defined as those maternities resulting in two births with identical, or immediately consecutive, dates of birth.

The odds of breast cancer in women who had delivered twins relative to other parous women (odds ratio, OR) was estimated by maximum likelihood using conditional logistic regression in the

*Received 20 September 1996*
*Revised 22 November 1996*
*Accepted 22 November 1996*

*Correspondence to:* M Murphy

Table Odds ratios (95% CI) of breast cancer in women who delivered twins relative to women who delivered single births only

| | Number of cases | Additional factors adjusted for | |
|---|---|---|---|
| | | None | Age at first birth and parity |
| All twin births | 64 | 0.70 (0.54–0.90) | 0.71 (0.55–0.91) |
| Unlike-sex twins | 17 | 0.72 (0.44–1.18) | 0.73 (0.44–1.19) |
| Like-sex twins | 47 | 0.69 (0.51–0.93) | 0.70 (0.52–0.94) |
| Twins as last birth | 43 | 0.68 (0.50–0.92) | 0.67 (0.49–0.91) |
| Twins not as last birth | 21 | 0.75 (0.48–1.17) | 0.81 (0.52–1.27) |

The women were matched on exact year of birth in all analyses.

computer package EPICURE (Preston et al, 1993). Approximate 95% confidence intervals (CIs) for the ORs were derived from the standard errors of the coefficients provided by the modelling procedure. Cases and control subjects were classified according to age at first birth in 18 categories (18 years or less, 19 ... 35+ years), interval since last birth in 11 categories (0, 1, 2 ... 10+ years) and parity defined as number of maternities in five categories (1, 2, 3, 4, 5+ maternities), where a multiple birth counts as one maternity. Adjusted ORs were estimated by fitting models that included factors representing these categorical variables.

### RESULTS

There were 4790 cases and 46751 controls available for analysis. Sixty-four of the cases and 895 of the control subjects had given birth to twins before the relevant date of diagnosis. The overall odds ratio for breast cancer in women who had delivered twins was 0.70 (Table). Additional adjustment for age at first birth and parity had very little effect on the estimate. Adjustment for time since last birth similarly had little effect (OR = 0.69, 95% CI 0.54–0.90). When restricting the analysis to twins of unlike sex (definitely dizygotic), or to twins which were the women's most recent birth, the ORs remain largely unaffected, although the confidence intervals widen because of the smaller number of twin mothers included.

### DISCUSSION

Our results provide evidence that women who deliver twins are at reduced risk of breast cancer relative to parous women who have not given birth to twins. The large data set used for the analysis comprised good quality information on breast cancer, birth history and some important confounding factors, although information about incomplete pregnancies (abortions, miscarriages) and other aspects of reproductive life and breast cancer risk were not available to us. The nature of the study design restricted our attention to women who were probably premenopausal when they developed breast cancer or were selected as control subjects. Our results change little with restriction to definitely dizygotic twin pregnancies, when examined according to whether the twins were the women's most recent birth or not, or on adjustment for age at first birth and parity. Two other case–control studies examining this issue also found a protective effect, while two did not (Jacobson et al, 1989; Nasca et al, 1992; Hsieh et al, 1994; Dietz et al, 1995). A protective effect was seen in one cohort study but not another (Wyshak et al, 1983; Albrektsen et al, 1995).

The largest of the case–control studies – the Cancer and Steroid Hormone (CASH) study – of nearly 4000 cases and frequency-matched control subjects aged 20–54 years (Jacobson et al, 1989) showed a protective effect of multiple births (OR 0.75, 95% CI 0.58–0.96). However, when examined according to birth order, the effect after adjustment was significant only when the multiple birth was the women's last birth (OR 0.60, 95% CI 0.43–0.85), but not for those before the last (OR 1.11, 95% CI 0.79–1.57); ORs for these two groups differing significantly. Dietz et al (1995) compared 5880 parous cases and 8217 control subjects under age 75 years, and found a non-significantly reduced risk (OR 0.94, 95% CI 0.75–1.17) after adjustment, but this effect was closer to ours when considering only those aged < 55 years (OR 0.83, 95% CI 0.57–1.22, personal communication). Nasca et al's (1992) report analysing pooled data from two studies totalling 1000 cases and an equal number of control subjects aged 20–54 years suggested a non-significantly increased risk (OR 1.10, 95% CI 0.67–1.81) after adjustment. Similarly, Hsieh et al's (1994) study of 1535 cases and 5038 control subjects aged under 55 years showed a non-significant adjusted increase in risk (OR 1.29, 95% CI 0.92–1.82).

The results of both Nasca et al (1992) and Hsieh et al (1994) for their study subjects aged > 55 years were similar in size and direction of the risk observed to their results for those aged < 55 years, but even closer to the null. Albrektsen et al (1995) found a non-significantly reduced risk (OR 0.89, 95% CI 0.73–1.09) after adjustment in a very large cohort study of parous Norwegian women aged under 56 years at diagnosis. Wyshak et al's (1983) smaller matched historical prospective study of incidence and mortality among 4000 mothers of dizygotic twins from the Connecticut twin register found a small non-significant increase in risk (relative risk 1.11, approximate 95% CI calculated from data in the paper 0.82–1.52). Wyshak et al's (1983) study will have included substantial numbers of post-menopausal breast cancer cases, but it is not possible to stratify their results by age at diagnosis.

Studies in which the twin pregnancies were likely to have occurred before the introduction of ovulation induction for subfertility (Wyshak et al, 1983; Hsieh et al, 1994) might produce different results from those more likely to include twin pregnancies of mixed origins (Jacobson et al, 1989; Nasca et al, 1992; Dietz et al, 1995; Albrektsen et al, 1995; and our own). This is to some extent evident. However, excluding the 10–11% of cases and control subjects whose pregnancies might have arisen from ovulation induction in the CASH study (Jacobson et al, 1989) had little effect on their results, as was also the case in the study of Nasca et al (1992). We cannot directly evaluate this in our study.

On balance, taking account of study sizes and effects demonstrated, the evidence so far suggests that in women aged less than 55 years, for whom there are more data available than at older ages, twinning is protective against breast cancer. Above age 55 years, the results are closer to, and compatible with, the null. It is possible that a protective effect may be modified by age at diagnosis. This protection may be caused by the additional hormonal changes during (any type of) twin pregnancy, or a hormonal state of 'dizygotic twin proneness' after menarche. The CASH investigators (Jacobson et al, 1989) invoked a role for alpha-fetoprotein combining with oestradiol to form an oestrogen receptor blocker to explain why the protective effect was restricted to last twin birth. In our study, the protective effect was apparent regardless of whether the twin birth was the women's last, but because the confidence intervals around the different estimates are wide, we cannot rule out a greater protective effect of last twin birth, as suggested also by the data of Albrektsen et al (1995). Increasing age at last full-term pregnancy has been positively associated with breast cancer risk (Kalache et al, 1993), and Hsieh et al (1994) suggested that the effect of the most recent twin pregnancy on risk varied with time since that pregnancy. In our study, however, the protective effect of twin birth was unaffected by adjustment for time since last birth. More detailed investigation of the effects of timing and type of twin birth on breast cancer risk by pooling results from the studies cited here might be valuable.

The hormonal profile in pregnancy or during menstrual life seems to us the most likely candidate, but other explanations, e.g. those involving alpha-fetoprotein or other genotype/phenotype characteristics of women who bear twins, might play a part (Wyshak, 1981; Murphy, 1995). Attention could now be paid to the ways in which mothers of (different types of) twins differ from other parous women, by comparing their menstrual, pregnancy and post-childbearing hormone levels, including progesterone. There is very little information on these levels outside pregnancy, and on whether pregnancy levels vary by type of twin. Currently, about half of all twin deliveries in England and Wales are 'natural' dizygotic conceptions, and a little more than a third are monozygotic (Murphy, 1995). Information about the exposure of mothers of twins to different levels of oestrogen and progesterone may provide further insight into the role played by these steroid hormones in breast cancer aetiology.

### ACKNOWLEDGEMENTS

We would like to thank Anders Ericson of Socialstyrelsen, Sweden whose cooperation made this study possible. Sophia Winters of the ICRF GPRG typed the manuscript. Mireille Broeders contributed to the analyses during a three-month placement at the London School of Hygiene and Tropical Medicine under the ERASMUS scheme. Jan Gunnarskog died tragically during the preparation of this manuscript.

### REFERENCES

Albrektsen G, Heuch I and Kvale G (1995) Multiple births, sex of children and subsequent breast cancer risk for the mothers: a prospective study in Norway. *Int J Cancer* **60**: 341–344

Bernstein L and Ross RK (1993) Endogenous hormones and breast cancer risk. In Breast Cancer, Epidemiology Review, Kelsey JL (ed.), pp. 48–65

Dietz AT, Newcomb PA, Storer BE, Longnecker MP and Mittendorf R (1995) Multiple births and risk of breast cancer. *Int J Cancer* **62**: 162–164

Hsieh CC, Lan SJ, Ekbom A, Petridou E, Adami HO and Trichopoulos D (1992) Twin membership and breast cancer risk. *Am J Epidemiol* **136**: 1321–1326

Hsieh CC, Goldman M, Pavia M, Trichopoulos D, Petridou E, Ekbom A and Adami HO (1994) Re: 'The relation between multiple births and maternal risk of breast cancer' and 'Multiple births and maternal risk of breast cancer' (letter). *Am J Epidemiol* **139**: 445–446

Jacobson HI, Thompson WD and Janerich DT (1989) Multiple births and maternal risk of breast cancer. *Am J Epidemiol* **129**: 865–873

Kalache A, Maguire A and Thompson SG (1993) Age at last full term pregnancy and risk of breast cancer. *Lancet* **341**: 33–36

Leon DA, Carpenter LM, Broeders MJM, Gunnarskog J and Murphy MFG (1995) Breast cancer in Swedish women: evidence of a dual effect of completed pregnancy before age 50. *Cancer Causes Control* **6**: 283–291

Murphy MFG (1995) The association of twinning with long-term disease. In *Multiple Pregnancy*, Ward RH and Whittle M (eds), pp. 14–29. RCOG Press: London

Nasca PC, Weinstein A, Baptiste M and Mahoney M (1992) The relation between multiple births and maternal risk of breast cancer. *Am J Epidemiol* **136**: 1316–1320

Preston DL, Lubin JH, Pierce DA and McConney ME (1993) *Epicure User's Guide*. Hirosoft International Corporation: Seattle

Short RV (1984) Testis size, ovulation rate and breast cancer. In *One Medicine*, Ryder OA, Byrd ML (eds), pp. 32–44. Springer-Verlag: Berlin

Wyshak G (1981) Reproductive and menstrual characteristics of mothers of multiple births and mothers of singletons only: a discriminant analysis. In *Twin Research 3: Twin Biology and Multiple Pregnancy. Progress in Clinical and Biological Research*, Volume 69A, Gedda L, Parisi P, Nance W (eds), pp. 95–105. Alan R Liss: New York

Wyshak G, Honeyman MS, Flannery JT and Beck AS (1983) Cancer in mothers of dizygotic twins. *J Natl Cancer Inst* **70**: 593–599

*© Cancer Research Campaign 1997*